# Testosterone enables growth and hypertrophy in fusion impaired myoblasts that display myotube atrophy: deciphering the role of androgen and IGF-I receptors 

**DOI:** 10.1007/s10522-015-9621-9

**Published:** 2015-11-04

**Authors:** David C. Hughes, Claire E. Stewart, Nicholas Sculthorpe, Hannah F. Dugdale, Farzad Yousefian, Mark P. Lewis, Adam P. Sharples

**Affiliations:** Stem Cells, Ageing & Molecular Physiology Unit, Research Institute for Sport and Exercise Sciences (RISES), School of Sport and Exercise Sciences, Liverpool John Moores University, Liverpool, UK; Department of Neurobiology, Physiology and Behavior, University of California, Davis, CA USA; Institute for Clinical Exercise and Health Science (ICEHS), University of the West of Scotland, Hamilton, Scotland, UK; National Centre for Sport and Exercise Medicine, School of Sport, Exercise and Health Science, Loughborough University, Loughborough, UK

**Keywords:** Testosterone, Akt, Satellite cell, Muscle, IGF-I, Aging, Androgens

## Abstract

We have previously highlighted the ability of testosterone (T) to improve differentiation and myotube hypertrophy in fusion impaired myoblasts that display reduced myotube hypertrophy via multiple population doublings (PD) versus their parental controls (CON); an observation which is abrogated via PI3K/Akt inhibition (Deane et al. 2013). However, whether the most predominant molecular mechanism responsible for T induced hypertrophy occurs directly via androgen receptor or indirectly via IGF-IR/PI3K/Akt pathway is currently debated. PD and CON C_2_C_12_ muscle cells were exposed to low serum conditions in the presence or absence of T (100 nM) ± inhibitors of AR (flutamide/F, 40 μm) and IGF-IR (picropodophyllin/PPP, 150 nM) for 72 h and 7 days (early/late muscle differentiation respectively). T increased AR and Akt abundance, myogenin gene expression, and myotube hypertrophy, but not ERK1/2 activity in both CON and PD cell types. Akt activity was not increased significantly in either cell type with T. Testosterone was also unable to promote early differentiation in the presence of IGF-IR inhibitor (PPP) yet still able to promote appropriate later increases in myotube hypertrophy and AR abundance despite IGF-IR inhibition. The addition of the AR inhibitor powerfully attenuated all T induced increases in differentiation and myotube hypertrophy with corresponding reductions in AR abundance, phosphorylated Akt, ERK1/2 and gene expression of IGF-IR, myoD and myogenin with increases in myostatin mRNA in both cell types. Interestingly, despite basally reduced differentiation and myotube hypertrophy, PD cells showed larger T induced increases in AR abundance vs. CON cells, a response abrogated in the presence of AR but not IGF-IR inhibitors. Furthermore, T induced increases in Akt abundance were sustained despite the presence of IGF-IR inhibition in PD cells only. Importantly, flutamide alone reduced IGF-IR mRNA in both cell types across time points, with an observed reduction in activity of ERK and Akt, suggesting that IGF-IR was transcriptionally regulated by AR. However, where testosterone increased AR protein content there was no increases observed in IGF-IR gene expression. This suggested that sufficient AR was important to enable normal IGF-IR expression and downstream signalling, yet elevated levels of AR due to testosterone had no further effect on IGF-IR mRNA, despite testosterone increasing Akt abundance in the presence of IGF-IR inhibitor. In conclusion, testosterones ability to improve differentiation and myotube hypertrophy occurred predominately via increases in AR and Akt abundance in both CON and PD cells, with fusion impaired cells (PD) showing an increased responsiveness to T induced AR levels. Finally, T induced increases in myotube hypertrophy (but not early differentiation) occurred independently of upstream IGF-IR input, however it was apparent  that normal AR function in basal conditions was required for adequate IGF-IR gene expression and downstream ERK/Akt activity.

## Introduction

The regulation of skeletal muscle mass is reliant on the balance between hypertrophy (e.g. protein synthesis/anabolism) and atrophy (e.g. protein breakdown/catabolism). A potential clinical intervention for promoting a positive net balance in the favor of protein synthesis is Testosterone (T) administration (Brodsky et al. 1996; Sheffield-Moore et al. 1999). Indeed, it has been suggested that testosterone replacement therapy can increase muscle strength and mass in various clinical populations, including patients with age related decline in muscle size and strength (sarcopenia) (Bhasin et al. 2000; Ferrando et al. 2002; Casaburi et al. 2004; Sattler et al. 2011; Dillon et al. 2012). In rodents, Serra et al. (2013b) observed T induced increases in skeletal muscle regeneration in both young and aged mice post cardiotoxin injury, which culminated in increased satellite cell activation and number. Further, testosterone-filled implants administered to young and old mice resulted in increases in gastrocnemius muscle weight and fiber cross-sectional area. The preservation of muscle mass was accompanied by decreases in myostatin and restoration of aged induced reductions in Akt activity and notch signaling (Kovacheva et al. 2010). Most recently, using murine heterochronic parabiotic models, testosterone administration in castrated old mice restored gastrocnemius muscle weight, muscle fiber cross-sectional area, and notch-1 expression (Sinha et al. 2014); observations that were not apparent when testosterone was absent. Interestingly, in humans Sinha-Hikim et al. (2006) observed increased myogenin expression with graded doses of T administered in community-dwelling older men, which was also accompanied by increased muscle size compared to baseline. A finding that was substantiated with T administration in frail elderly men where a prevention of aged related muscle loss occurred during a 6 month period compared to the placebo group (Atkinson et al. 2010). Finally, in terms of muscle atrophy, testosterone has been demonstrated to prevent muscle mass reductions in 2-month old castrated mice (Serra et al. 2013a), and restore normal transcript levels of myostatin and atrogenes (Atrogin-1 and MuRF1) following androgen deprivation (De Naeyer et al. 2014). Collectively these studies highlight the potential of T administration in restoring aged and atrophying muscle phenotypes to those of young adults. However, it is only recently that studies have begun to investigate the molecular role of testosterone in skeletal muscle cell growth, differentiation, and hypertrophy during periods of muscle loss with age (Wu et al. 2010; White et al. 2012; Serra et al. 2013b).

In vitro muscle cell studies have been conducted to provide potential mechanisms of Ts action independently of age (Wannenes et al. 2008; Wu et al. 2010; Serra et al. 2011; Sculthorpe et al. 2012; Basualto-Alarcón et al. 2013). The majority of these studies demonstrate increases in differentiation and myotube hypertrophy with T in muscle cell culture. T has also been co-incubated with inhibitors for the AR, IGF-IR, Akt and mTOR in a number of cell lines and primary cultures (Wannenes et al. 2008; Wu et al. 2010; Serra et al. 2011; Sculthorpe et al. 2012; Basualto-Alarcón et al. 2013; Deane et al. 2013) in order to elucidate the mechanisms of T induced muscle hypertrophy. However, these pathways were studied in isolation and there is currently no information about the most predominant mechanism of T induced growth either via a potential ‘indirect’ increase in IGF-I gene expression and associated signaling, which has more recently been suggested to be important in regulating T induced hypertrophy (Serra et al. 2011; Sculthorpe et al. 2012; White et al. 2012), versus the traditionally ‘direct’ androgen receptor mediation (Lee 2002; Altuwaijri et al. 2004; Wannenes et al. 2008). Furthermore, we have recently shown that PI3K inhibitor (LY294002) attenuates the testosterone induced increases in differentiation and myotube hypertrophy, suggesting that IGF-I/IGF-IR and downstream signaling are important in mediating testosterones action (Deane et al. 2013).

In order to elucidate the combined pathways that control both muscle cell adaptation and ageing, our recent studies have highlighted two myoblast models to study reduced differentiation capacity and myotube atrophy (Sharples et al. 2010, 2011). The first model investigated parental mouse C2 myoblasts versus their daughter C2C12 cells (subclone). Despite shared origins, the parental C2 cells displayed slower and diminished differentiation profiles compared to the daughter C2C12 cells and were also more susceptible to TNF-α-induced inhibition of differentiation and induction of cell death (Sharples et al. 2010, 2015). The second model utilized C_2_C_12_ cells that had undergone 58 population doublings (PD) versus parental control cells (CON), which have undergone no doublings relative to the PD cells (Sharples et al. 2011). Like C2 cells in the first model and relative to the CON cells, the PD cells also displayed impaired differentiation in monolayer (Sharples et al. 2011) and reduced myotube hypertrophy in both monolayer (Deane et al. 2013) and bioengineered three-dimensional (3D) culture systems (Sharples et al. 2012). PD cells also had a reduced number of cells exiting the cell cycle in G1 (pre-requisite for fusion), with corresponding decreases in gene transcripts controlling fusion such (myoD, myogenin, IGF-I) and reduced activation of Akt with parallel increases in fusion inhibitory factors such as; IGFBP5 mRNA and JNK activation (Sharples et al. 2011) versus control cells. Further basally, PD cells had higher myostatin (negative regulator of muscle mass) and lower mTOR gene expression (required for differentiation/myotube hypertrophy) in mature myotubes (Deane et al. 2013). These adaptations were confirmed in 3D bioengineered muscle systems (Sharples et al. 2012) where myostatin mRNA increased as did TNF-α transcript expression with reductions in peak force generation by the cells on attachment to the 3D matrix (Sharples et al. 2012). Importantly, PD cells therefore displayed  a similar gene transcript (decreased myoD, myogenin, IGF-I and increased TNF-α and myostatin), signaling processes (e.g. reduced phosphorylated Akt) and morphology (impaired differentiation and myotube atrophy) to that previously observed following replicative aging in isolated human muscle cells (Bigot et al. 2008), in human/rodent cells isolated from aged individuals (Lees et al. 2006; Bigot et al. 2008; Leger et al. 2008; Lees et al. 2009; Pietrangelo et al. 2009) and from that observed in aged bioptic material (Welle et al. 2003; Leger et al. 2008). Critically, in this model the reduction in fusion in PD cells was not associated with senescence as there was no reduction in S/G2 phase cell cycle progression and the doubling times of the cells following multiple population doublings was not altered (Sharples et al. 2011). Thus, this multiple population doubled cellular model can be used as a representative model to investigate mechanisms of impaired differentiation and myotube atrophy, phenotypes similar to those observed in aged muscle cells. Previous work with this model has also demonstrated that T administration (100 nM) has been observed to improve impaired differentiation and myotube hypertrophy in PD cells to levels observed in control cells under basal conditions (Deane et al. 2013).

Consequently, as the primary signaling mechanisms for testosterone induced hypertrophy requires further investigation, and the mechanisms of T induced hypertrophy with age have yet to be fully determined, the aim of the current studies were to investigate: (1) The predominant pathway (androgen receptor vs. the IGF-I receptor and associated signaling ERK, Akt) that mediated the hypertrophic effects of testosterone using specific substrate competitors for AR and IGF-IR (flutamide and picropodophyllin, respectively) independently, and combination in the absence or presence of testosterone, and to investigate, (2) the role of these pathways in T induced differentiation and myotube hypertrophy in muscle cells that are fusion impaired and display characteristics of myotube atrophy (PD cells) versus relevant controls. It was hypothesized that the inhibition of the AR and IGF-I receptor would both impair testosterone induced increases in myotube differentiation and hypertrophy with PD cells rendered more susceptible versus CON cells.

## Methods

### Cell culture

Murine C_2_C_12_ (Yaffe and Saxel 1977; Blau et al. 1985) (ATCC, Rockville, MD, USA) skeletal muscle myoblasts were seeded at 80,000 cells/ml in 2 ml growth media (GM) per well. GM was made up of: Dulbecco’s modifed eagle’s medium, DMEM (Sigma, Dorset, UK. Cat no. D6429), 20 % fetal bovine serum (FBS) (PAA, Somerset, UK) and  1 % PenStrep (Invitrogen, Paisley, UK) Wells were coated with 0.2 % porcine gelatin (Sigma, Dorset, UK) using 6-well plates (Fisher Scientific, Loughborough, UK) and grown in a humidified 5 % CO_2_ atmosphere at 37 °C. Population doubled (PD) cells and their parental controls (CON) were developed as detailed by Sharples et al. (2011, 2012). Briefly, PD cells were subjected to 58–60 population doublings vs. the parental CON cells, that have undergone no doublings relative to the PD cells (Sharples et al. 2011). Further, the PD cells have reduced differentiation and display myotube atrophy at 72 h (Sharples et al. 2011) and 7 days (Deane et al. 2013) versus the parental CON cells. Once confluent, the myoblasts were transferred to low serum media/differentiation media (DM; composed of: DMEM, 2 % horse serum (HS), 1 % Penstrep), which promotes the spontaneous fusion of the myoblasts into multinucleated myotubes (Blau et al. 1985). Cells were incubated in DM for 30 min at 37 °C in a 5 % CO_2_ atmosphere with this period of equilibration denoted as the 0 h time point. Cells at the time points of 72 h and 7 days (early and late muscle differentiation respectively) were fixed for subsequent morphology analyses, mRNA extracted for gene expression studies via reverse transcription quantitative real-time polymerase chain reaction (rt-qRT-PCR) and lysed for cell signaling analyses via SDS PAGE and Western blot (all detailed below).

### Cell treatments

All treatments were administered in DM described above. Initial dose responses were initially performed for AR inhibitor (flutamide/F 20 and 40 μm) and IGF-IR inhibitor; picropodophyllin/PPP 30, 90 and 150 nM) to ascertain the most effective treatment (e.g. via optimum reductions in total AR content for flutamide and via reduction in Akt and ERK activation for IGF-IR inhibition). At 40 µM Flutamide reduced AR by over 70 % in CON and in PD cells after 72 h and 150 nM Picropodophyllin reduced Akt by an average of ~43 % and ERK1/2 by 32 % across both CON and PD cells over both 30 min and 72 h post DM transfer. Following optimisation; the treatments comprised of a vehicle control (differentiation media/DM alone plus DMSO at a concentration of 0.01 %), testosterone (100 nM as used in Deane et al. 2013), flutamide (40 μM), picropodophyllin (150 nM), co-incubations of the inhibitors with T (i.e. T + F, T + PPP, T + F + PPP). For both results and figure legends the following nomenclature for dosing conditions will be used: Control (DM conditions above), testosterone (T), flutamide (F), and picropodophyllin (PPP) respectively. All treatments were added at 0 h and existing media was further supplemented with 1 ml of fresh media at 72 h for cells that were maintained to 7 days.

### RNA isolation

Six-well plates for each time point (0, 72 h and 7 days) were washed with 1 ml/well phosphate buffer saline (PBS) and extracted for RNA using 300 μl TRIzol™ Reagent/well (Sigma, Poole, UK). Total RNA was extracted by following manufacturer’s instructions. RNA concentration and purity were assessed through UV spectroscopy at ODs of 260 and 280 nm, using the Nanodrop spectrophotometer 2000c (Fisher, Rosklide, Denmark). Samples had 260:280 ratios between 1.8 and 2.2.

### Primer design

Primer sequences (Table 1) were identified using Gene (NCBI, www.ncbi.nlm.nih.gov/gene) and designed using both web-based OligoPerfect™ Designer (Invitrogen, Carlsbad, CA, USA) and Primer-BLAST (NCBI, http://www.ncbi.nlm.nih.gov/tools/primer-blast). Sequence homology searches ensured specificity with no un-intended targets identified. Three or more GC bases in the last five bases at the 3′ end of the primer were avoided. Secondary structure interactions (hairpins, self-dimer and cross dimer) within the primer were also avoided. All primers ranged between 18 and 23 bp and amplified a product between 125 and 197 bp. GC content was between 36.3 and 60.0 % (Table 1). Primers without the requirement of further purification were purchased from Sigma (Poole, UK).

**Table 1:** Primer sequences for genes of interest

Gene	Primer sequence (5′–3′)	Ref. sequence number	Amplicon length (bp)	GC% content
AR	F:GCCTCCGAAGTGTGGTATCC R:CCTGGTACTGTCCAAACGCA	NM_013476.3	138	60 55
IGF-IR	F:CTACCTCCCTCTCTGGGAATG R:GCCCAACCTGCTGTTATTTCT	NM_010513	185	47.4 47.4
MyoD	F:CATTCCAACCCACAGAAC R:GGCGATAGAAGCTCCAA	NM_010866	125	59.7 50
Myostatin	F:TACTCCGAATAGAAGCCATAA R:GTAGCGTGATAACGTCATC	NM_010834	194	36.3 45
Myogenin	F:CCAACTGAGATTGTCTGTC R:GGTGTTAGCCTTATGTGAAT	NM_031189	173	47.3 40
RP-IIb	F:GGTCAGAAGGGAACTTGTGGTAT R:GCATCATTAAATGGAGTAGCGTC	NM_153798.1	197	50 44.4

### Reverse transcription quantitative real time polymerase chain reaction (rt-qRT-PCR)

Seventy ng RNA/sample was reverse transcribed and amplified using QuantiFast™ SYBR^®^ Green RT-PCR one-step kit on a Rotogene 3000Q (Qiagen, Crawley, UK) supported by Rotogene software (Hercules, CA, USA). The rt-qRT-PCR was performed as follows: 10 min, 50 °C (reverse transcription), 5 min 95 °C (transcriptase inactivation and initial denaturation), followed by: 10 s, 95 °C (denaturation), 30 s, 60 °C (annealing and extension) for 40 cycles. Following completion, melting curve analyses were performed to exclude primer-dimer, contamination and non-specific amplification. All melt curves confirmed that single peak melt temperatures occurred for each gene of interest. Relative mRNA expression was quantified for myogenin, MyoD, AR, IGF-IR and myostatin (Table 1) using the comparative Ct (^ΔΔ^ct) method (Livak and Schmittgen 2001; Schmittgen and Livak 2008; Sharples et al. 2011) against a stable reference gene of polymerase (RNA) II polypeptide B (RP-IIb) (combined stable Ct value for all runs across experimental conditions 17.36 ± 1.03) and calibrator of CON cells at 0 h.

### SDS PAGE and immunoblotting

At the relevant time points cells were washed twice in 1 ml/well PBS and lysed in 300 µl/well (six well plates) using 10 mM TrisHCl, 5 mM EDTA, 50 mM sodium chloride, 30 mM sodium pyrophosphate, 50 mM sodium fluoride, 100 µM sodium orthovanadate, 1 mM PMSF, 1 % Triton X-100 and protease inhibitor tablets (Roche Diagnostics Limited, UK). Thirty µg of protein per cell lysate was added to sample buffer (5× Laemmili Buffer; 1 M Tris–HCl pH 6.8, SDS, Glycerol, dH_2_O, gel loading solution (Sigma Aldrich, Poole, UK) and β-mercaptoethanol). Molecular weights were determined by the incorporation of 10 µl rainbow molecular weight protein standards purchased from Sigma-Aldrich (Color Burst Electrophoresis Marker, Sigma, Poole, UK). The samples were boiled at 90 °C for 5 min before loading onto the gel with a 20 μl loading tip (Fisher Scientific, Loughborough, UK).

Gels were made up of a 10 % resolving gel (30 % acrylamide 1 % BIS, 1.5 M Tris base pH 8.9, 0.1 M EDTA pH 7.4, 10 % SDS, 0.1 % (w/v) APS and 0.1 % (v/v) TEMED) and a 5 % stacking gel (30 % acrylamide 1 % BIS, 54 mM Tris HCl pH 6.8, 0.1 M EDTA pH 7.4, 0.1 % (w/v) SDS, 0.1 % (w/v) APS and 0.1 % (v/v) TEMED). Samples were electrophoresed at 200 volts until proteins migrated to the bottom of the gels. The proteins were transferred using a semi dry technique (Trans blot turbo transfer system; Bio-Rad Laboratories, Inc. Hercules, CA, USA) to the nitrocellulose membrane for use in immunoblotting for 30 min. Immunoblotting was performed using a fast western supersignal west pico rabbit substrate kit (Pierce, Rockford, IL, USA). The western blotting was performed based on manufacturers instructions. Membranes were incubated in primary antibodies (New England Bio Labs, Santa Cruz, USA) for the following proteins: p-Akt (ser473) cat no. 4058, p-ERK1/2 (Thr202/Tyr204) cat no. 9101, total Akt cat no. 9272, total ERK1/2 cat no. 9102 all at a concentration of 1:1000, total AR (1:500) cat no. SC-816, and GAPDH (loading control; 1:4000) cat no. 5174, were diluted in an antibody diluent liquid (contained in the fast western supersignal pico rabbit substrate kit described above) and incubated overnight at 4 °C. Detection of proteins was achieved using enhanced chemiluminescence (ECL) with a West Pico Supersignal kit (Pierce, Rockford, IL, USA). The total and phosphorylated proteins were assessed relative to the GAPDH that was unchanged between conditions. Total AR, p-Akt and GAPDH were analysed on the same membrane due to distinct molecular weights. Phosphorylated ERK and GAPDH were also analyzed on the same membranes. Membranes were then stripped and re-probed for total ERK and Akt. Although proteins (p-Akt and p-ERK1/2) were analyzed on separate membranes between cell types i.e. (CON vs. PD, when mean ± SD were calculated globally for the arbitrary units (following detection), these were found to be non-significant between cell types (e.g. p-ERK1/2 72 h: CON 0.11 ± 0.02 vs. PD 0.16 ± 0.03; P = NS). Therefore comparisons between cell types were enabled. For the total AR protein, the same exposure times and batch of antibodies were selected for densitometry analysis allowing for comparison between CON and PD cell types.

### Morphology

**Fig. 1:**
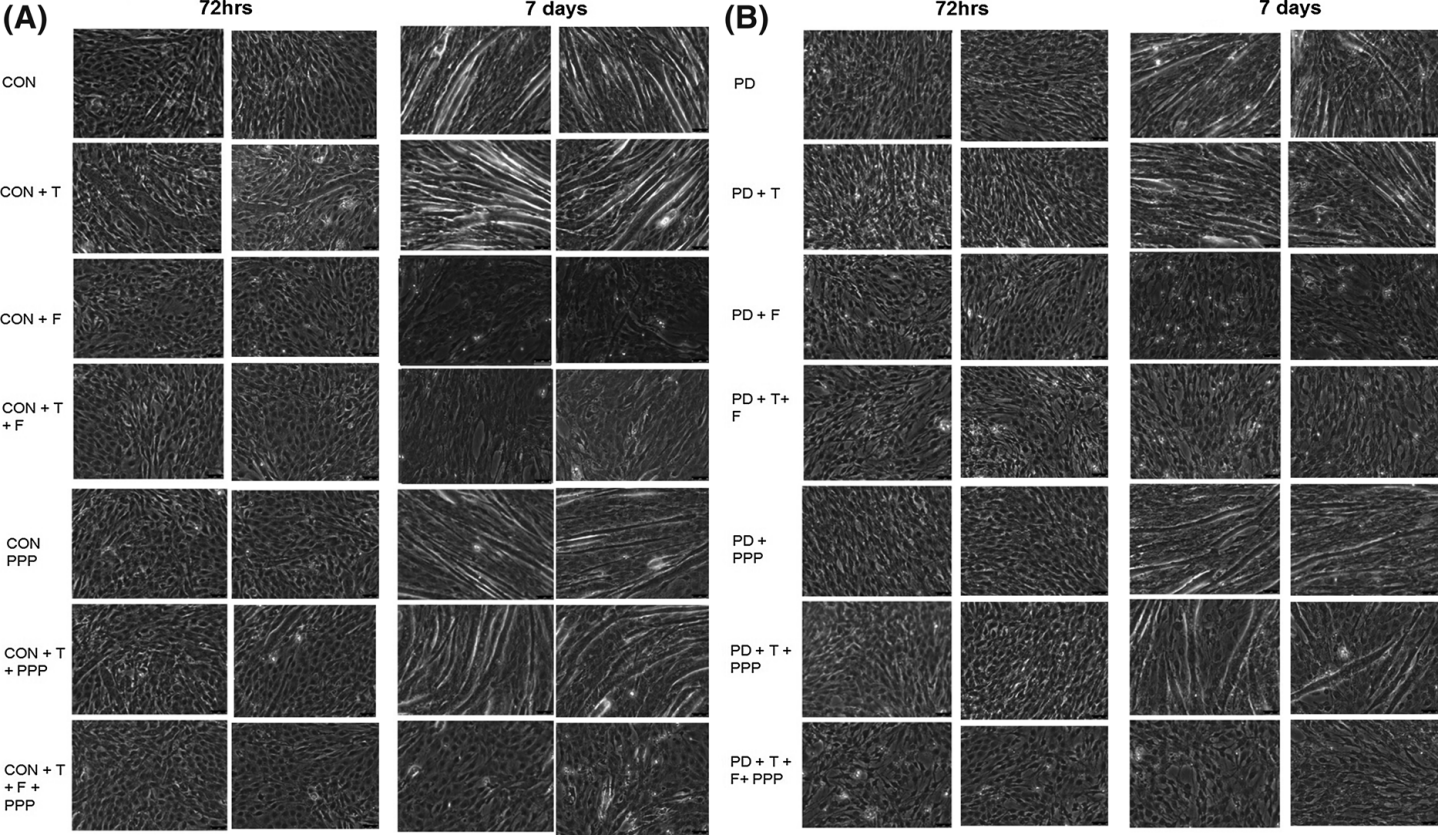
Representative light microscope (×20) images for CON (*Panel a*) and PD (*Panel b*) myotube morphology after 72 h and 7 days culture with inhibitor treatments (*DM* control, *T* testosterone, *F* flutamide, *PPP* picropodophyllin)

Cells were fixed by media being aspirated from the wells and replaced with 1 ml PBS and 0.5 ml of methanol/0.5 ml acetone ice-cold per well for 10 min and then 1 ml of acetone/1 ml of acetone per well for a further 10 min. PBS (2 ml/well) was added after removal of the methanol/acetone mix and plates were stored in 4 °C until further analysis. Cells were stained for desmin and nuclei using 1:200 desmin polyclonal rabbit anti-human (also reacts with mouse) antibody (Abcam, Cambridge, UK), 1:200 anti-rabbit TRITC secondary antibody (Sigma, St Louis, USA) and 1:2000 Sytox Green (Life Technologies, CA, USA) nuclear stain. The blocking/permeabilisation solution consisted of 1× TBS (pH 8.5), 5 % goat serum (Sigma Aldrich, Poole, UK) and 0.2 % Triton-X 100 (Fisher Scientific, Loughborough, UK). The antibody solutions consisted of 1× TBS, 2 % goat serum and 0.2 % Triton-X 100 plus either the primary or secondary antibody at concentrations described above. Sytox green was diluted in double distilled water. Myotubes were defined as 3+ nuclei within a cellular structure so to rule out cells undergoing mitosis. A total of 30 images per condition were captured on ×10 magnification fluorescent microscope (DM6000 FS, Leica, Germany). The microscope was setup to take separate images of desmin (yellow light), nuceli (green light) and a light microscope image of the same acquisition area (of which representative light microscope images are depicted in Fig. 1). Images were analysed using Image J (Java) software (National Institutes of Health, USA). Morphology was assessed by determination of myotube diameter, number of myotubes per view, mean number of nuclei per myotube per field of view. Myotube diameter (μm) was determined by measuring the diameter of three equidistant points on each myotube (left end, middle, right end) and determining the mean of the three values as previously described (Stevenson et al. 2005; Trendelenburg et al. 2009; Deane et al. 2013).

### Statistical analysis

Experiments were performed in duplicate, with three separate repeats (n = 3). Data are presented as Mean ± SD unless stated otherwise. Gene expression and morphology data was assessed using a mixed three-way (2 × 6 × 2) factorial ANOVA for interactions between time (72 h and 7 days), treatments (DM, T, F, PPP, T + F, T + PPP T + F + PPP) and cell types (CON and PD). Bonferroni post hoc analyses were then performed to establish where differences lay. A one way ANOVA was performed for western blots analyses to compare the effect of treatments between each cell type at 72 h and 7 days. A *P* value of <0.05 was considered statistically significant. All statistical analyses were performed using SPSS version 19 (IBM, Armonk, NY, USA) and Graph Pad Prism Software (San Diego, USA).

## Results

### AR (flutamide) and IGF-IR (picropodophyllin) inhibitors on testosterone-induced hypertrophy

**Fig. 2:**
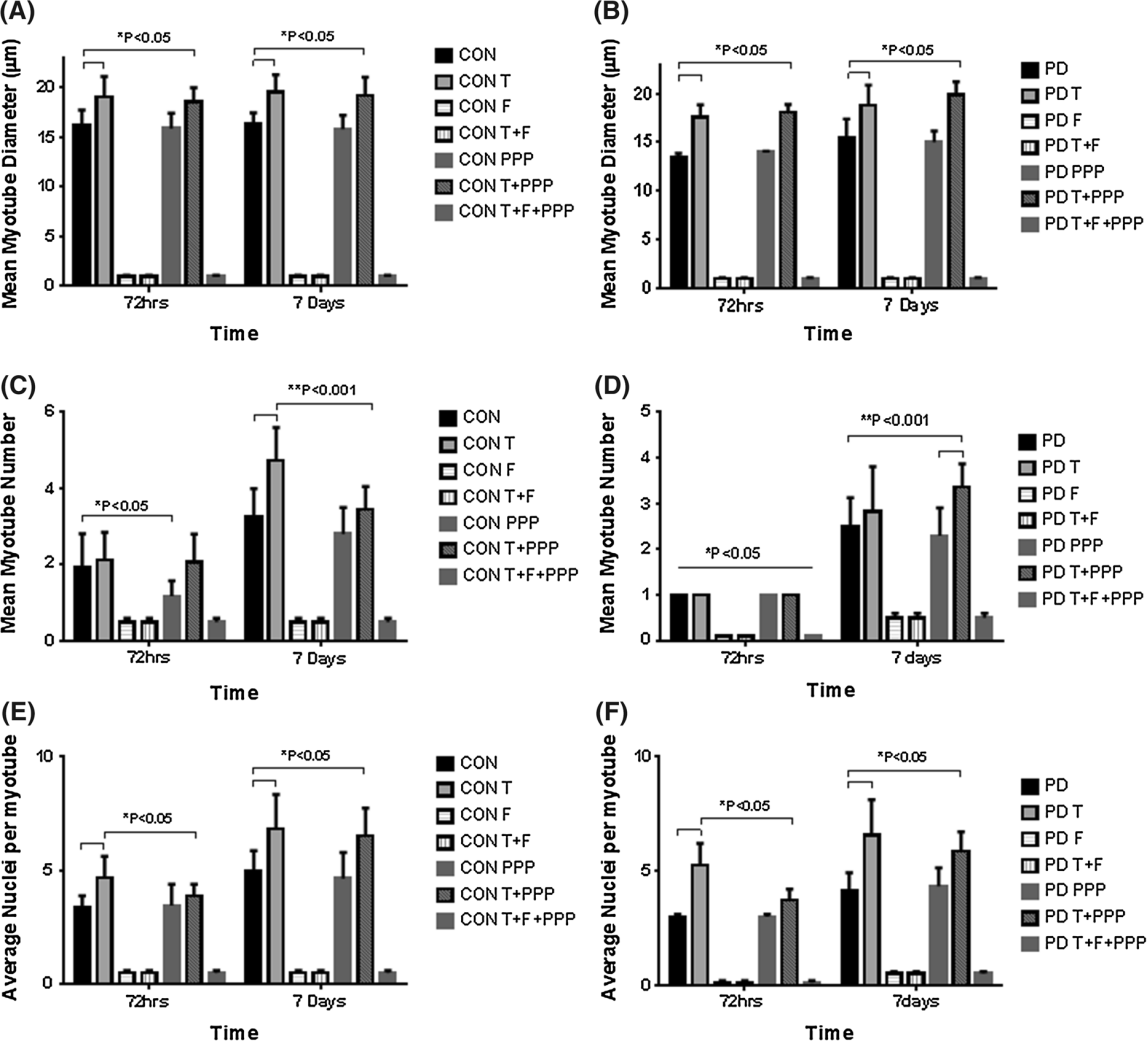
The effect of testosterone administration and co-incubations of flutamide (F) and Picropodophyllin (PPP) on myotube formation and hypertrophy. Testosterone was rendered inactive in its ability to promote differentiation in CON and PD muscle cells following its co-incubation with AR inhibitor flutamide, resulting in no quantifiable myotubes being observed at any time point. **a** In CON cells, testosterone significantly increased myotube diameter at 72 h and 7 days exposure. The co-incubation of testosterone with Picropodophyllin did not abrogate testosterones increases in myotube diameter at either time point (*P ≤ 0.05). **b** A similar trend occurred in PD myoblasts with testosterone treatment significantly increasing myotube diameter compared to un-treated cells (*P ≤ 0.05). The observed increases in testosterone induced hypertrophy continued at 7 days treatment (**P ≤ 0.05). The addition of PPP had no effect on altering the observed increases in hypertrophy at either time point in PD myoblasts. **c** The addition of PPP alone significantly reduced myotube number in CON myoblasts after 72 h (*P ≤ 0.05). Testosterone significantly increased myotube number after 7 days exposure (**P ≤ 0.001) in CON myoblasts, but the addition of PPP somewhat attenuated this increase. **d** Significant difference (*P < 0.05) between PD treatments at 72 h and 7 days for myotube number. The myotube number at 7 days in PD myoblasts was increased with the co-incubation of testosterone and PPP compared to untreated and PPP treated cells (**P ≤ 0.001). **e**, **f** Testosterone significantly increased the average number of nuclei per myotube in both CON and PD cells after 72 h (*P < 0.05) and 7 days (**P < 0.05) compared to untreated cells. The co-incubation of picropdophyllin with testosterone did not negate the observed increases in nuclei per myotube in either cell type after 7 days treatment. Values presented as Mean ± SD

Firstly, here we confirm from previous studies (Sharples et al. 2011, Deane et al. 2013) that myotube number is significantly reduced at 72 h and 7 days in PD versus CON cells (72 h CON 1.95 ± 0.86 vs. PD 1.0 ± 0; 7 days CON 3.27 ± 0.72 vs. PD 2.50 ± 0.62; P < 0.05; Fig. 2c, d) as was nuclei per myotube (7 days CON 4.93 ± 0.92 vs. PD 4.14 ± 0.69; P < 0.05; Fig. 2e, f). Myotube diameter was also significantly reduced at 72 h between CON and PD cells (CON 15.88 ± 1.55 vs PD 13.40 ± 0.47, P < 0.05; Fig. 2a, b) but not at 7 days (CON 15.81 ± 1.40 vs PD 15.52 ± 1.89; P < 0.05, Fig. 2a, b). Therefore, PD cells have reduced myotubes at both 72 h and 7 days that are less hypertrophied up to 72 h resulting in less nuclei per myotube by 7 days. Testosterone administration alone resulted in increases in differentiation (myotube number) and myotube hypertrophy indices (diameter and average nuclei per myotube) in both CON and PD cells compared to untreated myoblasts after 72 h and 7 days culture. These observations also confirm our previous published findings highlighting the role of testosterone administration in improving impaired myotube hypertrophy in the PD cell types (Deane et al. 2013). However, T was rendered inactive in its ability to promote differentiation in CON and PD muscle cells following its co-incubation with AR inhibitor flutamide, resulting in no quantifiable myotubes being observed (Fig. 1), suggesting that the successful binding of testosterone to the AR is fundamental in the above morphological processes. The IGF-IR inhibitor alone, PPP, significantly decreased myotube number compared to DM conditions in CON myoblasts after 72 h (CON DM 1.95 ± 0.86 vs. CON + PPP 1.18 ± 0.40; P ≤ 0.05, Fig. 2c). Although after 7 days there was still a mean decrease in myotube number present with PPP administration, this was not statistically significant (CON DM 3.27 ± 0.72 vs. CON + PPP 2.80 ± 0.70; P = NS, Fig. 2c). The co-incubation of PPP with testosterone abrogated the T induced increases in myotube number after 7 days exposure in CON cells (CON + T 4.73 ± 0.87 vs. CON + T + PPP 3.45 ± 0.60; P ≤ 0.05, Fig. 2c). In terms of myotube diameter; in CON cells, there was no significant effect at 72 h and 7 days between treated and un-treated myoblasts with PPP (P = NS, Fig. 2a). Although, PPP impaired T induced myotube number increases, the co-incubation of testosterone and PPP still resulted in T induced myotube hypertrophy evident via continued increases in myotube diameter at each time point (72 h CON + T 19.04 ± 2.07 vs. CON + T + PPP 18.54 ± 1.45: 7 days CON + T 19.54 ± 1.77 vs. CON + T + PPP 19.23 ± 1.82; P = NS, Fig. 2a). Additionally, PPP alone did not significantly alter mean nuclei per myotube in CON myoblasts after 72 h and 7 days compared to DM treated cells (P = NS). Finally, the co-incubation of testosterone with PPP did not abrogate the significant increases in mean nuclei per myotube with testosterone administration alone at any time point (72 h CON + T 4.68 ± 0.92 vs. CON + T + PPP 3.86 ± 0.52: 7 days CON + T 6.80 ± 1.51 vs. CON + T + PPP 6.50 ± 1.22; P = NS).

In PD cells, PPP addition resulted in no significant differences in myotube number compared to un-treated cells after 7 days exposure (P = NS, Fig. 2c). A significant difference was detected between PPP alone and the co-incubation of PPP plus testosterone for myotube number in PD myoblasts after 7 days culture (PD + PPP 2.29 ± 0.61 vs. PD + T 2.82 ± 0.98; PD + T + PPP 3.36 ± 0.50; P ≤ 0.01, Fig. 2c). For myotube diameter, the presence of PPP had no effect on testosterone-induced hypertrophy in PD myoblasts at any time point studied (72 h PD + PPP 14.00 ± 0.10 vs. PD + T + PPP 18.06 ± 0.83: 7 days PD + PPP 15.03 ± 1.14 vs. PD + T 18.79 ± 2.10; PD + T + PPP 19.87 ± 1.35; P ≤ 0.01, Fig. 2b). In addition, the co-incubation of PPP with testosterone had no effect on abrogating the increases in mean nuclei per myotube with testosterone administered alone (PD + T 6.58 ± 1.54 vs. PD + T + PPP 5.85 ± 0.86; P = NS). Overall, in terms of myotube morphology, it appears that testosterone-induced hypertrophy is not mediated via the IGF-IR in both cell types as testosterone still induced appropriate increases in myotube hypertrophy even in the presence of the IGF-IR inhibitor. The data do, however, suggest that Testosterones effects are fully inhibited in the presence of flutamide, the AR inhibitor, in both cell types, providing initial evidence for the more predominant role of successful binding of T to AR versus a secondary effect via the IGF-IR in testosterone mediated myotube hypertrophy.

### The effect of testosterone administration on androgen receptor (AR) protein abundance

**Fig. 3:**
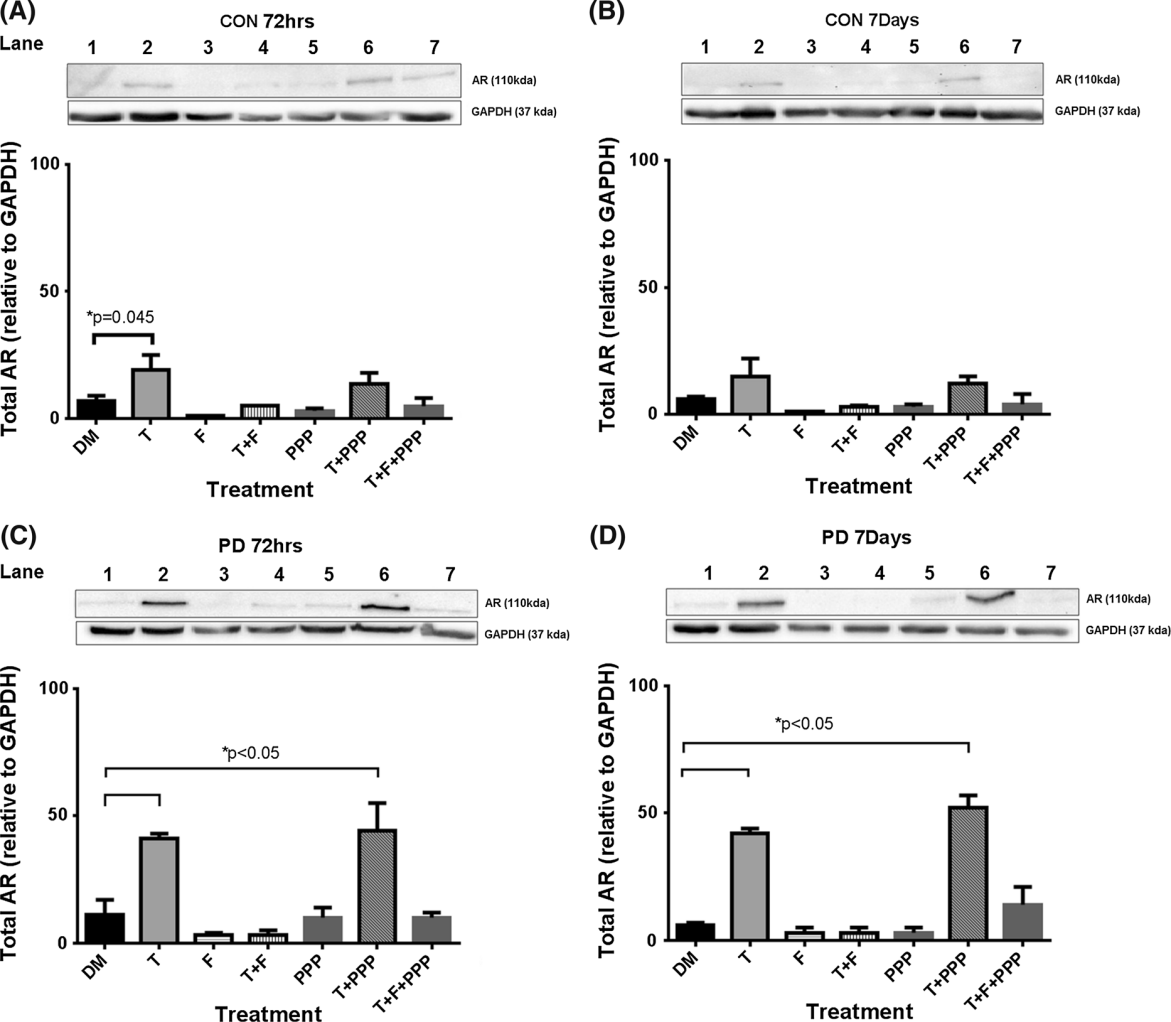
The effect of testosterone and co-incubation treatments on total AR protein levels in CON (**a**, **b**) and PD (**c**, **d**) myoblasts after 72 h and 7 days. **a** The addition of a testosterone stimulus alone significantly increased AR levels in CON cells after 72 h culture (*P = 0.045). **c** Testosterone treatment significantly increased AR protein levels, even in the presence of Picropodophyllin for PD myoblasts (*P < 0.05). Flutamide administration inhibited testosterone induced increases in AR protein levels. **d** Testosterone treatment significantly increased AR protein levels, even in the presence of Picropodophyllin (*P < 0.05). The presence of flutamide abrogated these increases in AR protein levels under a testosterone stimulus. Values presented as Mean ± SEM

A testosterone dose (100 nM) was sufficient to increase AR protein levels in both CON and PD myoblasts for each time point. In CON myoblasts, testosterone significantly increased AR protein levels (arbitrary units) alone after 72 h (CON + T 19 ± 6 vs. CON DM 7 ± 2; P = 0.045. Figure  3a and remained elevated (although not statistically significant) after 7 days (CON + T 15 ± 7 vs. CON DM 6 ± 1; P = NS. Fig. 3b). The presence of AR protein with flutamide administration alone seemed to be completely abolished for both 72 h and 7 day blots (Fig. 3a, b respectively). It was interesting however, that in the testosterone plus IGF-IR inhibitor (PPP), AR total protein was seemingly restored back to basal levels suggesting that testosterone administration (similar to T alone) induces increases in AR, as expected, that are not dependent on IGF-IR (72 h CON T 18 ± 6 % vs. CON T + PPP 13.5 ± 4.5 %; 7 days CON T 15 ± 7 % vs. CON T + PPP 12 ± 3 %; P = NS).

**Fig. 4:**
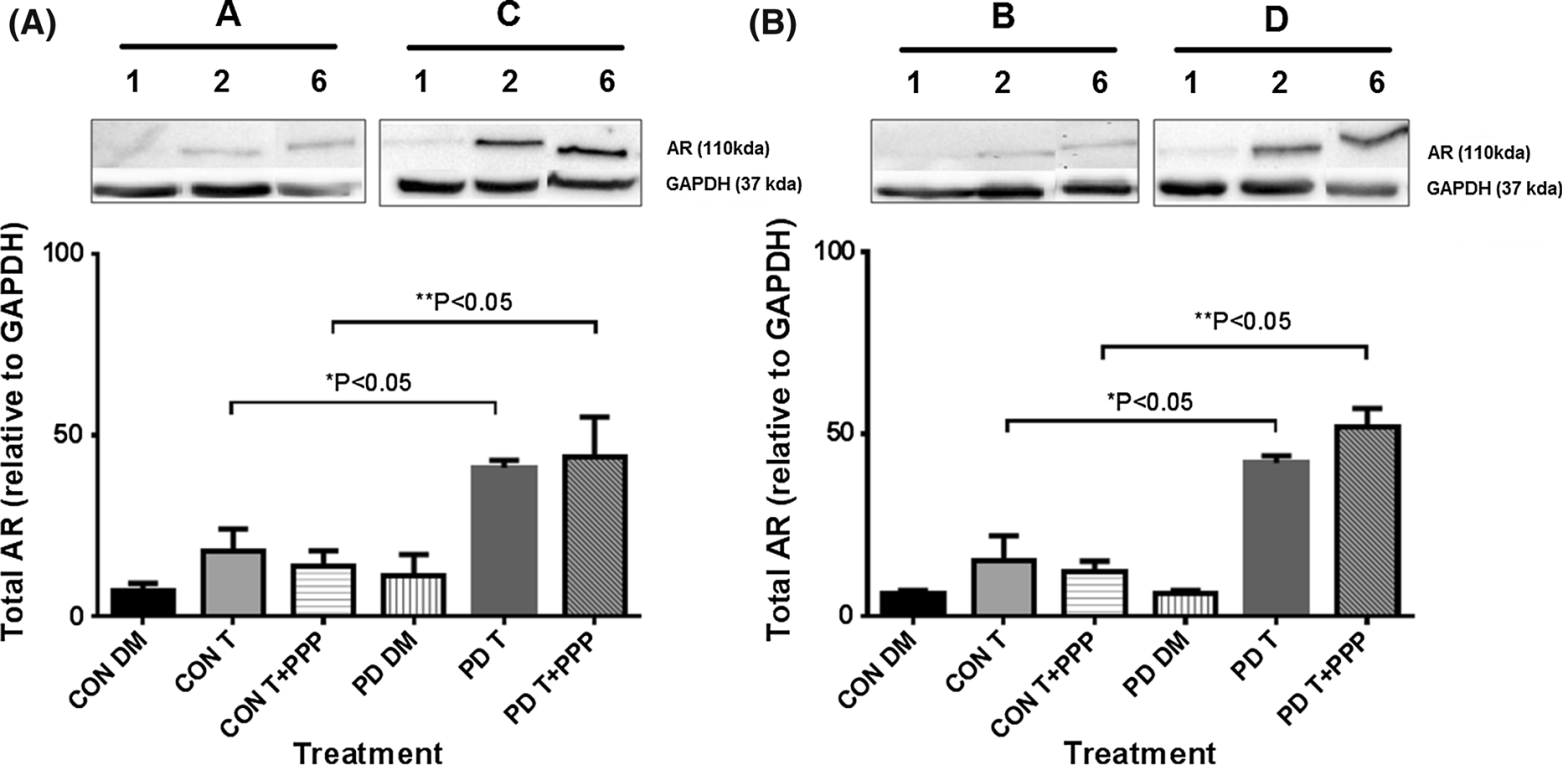
Comparison between total AR levels in CON and PD myoblasts exposed to testosterone alone and in combination with picropodophyllin after 72 h (**a**) and 7 days (**b**). Please note, Representative blot images are summarized from Fig. 3 (figure number and lane number where these are taken from is depicted above the blot bands) in order to enable comparisons with CON and PD cells visually. This is because CON and PD cell types were analyzed on separate membranes with the same exposure time. Densiometry was comparable between membranes to allow comparisons between CON and PD cells where it was confirmed that GAPDH (detected at the time on the same membrane) density values were not significantly different across membranes (P > 0.05). Data in figures is representative of n = 3. **a** Testosterone significantly increased AR levels in PD myoblasts versus CON myoblasts after 72 h (*P ≤ 0.05). These increases in AR remained in the PD cells co-incubated with picropodophyllin compared to the same treatment in CON cells (**P ≤ 0.05). **b** At 7 days culture, the same pattern was evident, where by Testosterone alone (*P ≤ 0.05) and when co-incubated with picropodophyllin (**P ≤ 0.05) significantly increased AR levels compared with the same treatments in CON myoblasts. Values presented as Mean ± SEM

In the PD myoblasts, testosterone significantly increased the AR protein levels, even in the presence of Picropodophyllin after 72 h, compared  to basal control (PD + T 41 ± 2 %; PD T + PPP 44 ± 11 % vs. PD DM 11 ± 6 %; P ≤ 0.05, Fig. 3c) and at 7 days (PD + T 42 ± 2 %; PD T + PPP 52 ± 5 % vs. PD DM 6 ± 1 %; P ≤ 0.05 Fig. 3d). Flutamide significantly abrogated testosterone induced increases in AR protein levels after 72 h (PD + T 41 ± 2 %vs. PD T + F 3 ± 2 %; P ≤ 0.05, Fig. 4c) and 7 days (PD + T 42 ± 2 % vs. PD T + F 3 ± 2 %; P ≤ 0.05, Fig. 3d). Furthermore, the presence of flutamide in combination with testosterone and PPP, decreased AR protein levels compared to testosterone incubated with PPP (72 h PD T + PPP 44 ± 11 % vs. PD T + F + PPP 10 ± 2 %: 7 days PD T + PPP 52 ± 5 % vs. PD T + F + PPP 14 ± 7 %; P ≤ 0.05, Fig. 3c, d) at both time points. Together with morphological findings these data provide evidence that testosterone increases AR total protein and this increase is abrogated in the presence of flutamide but not PPP, again suggesting an important role for AR and not the IGF-IR/associated signaling in testosterones impact on the morphological parameters measured in CON and PD cells.

Finally, after 72 h there were no significant differences in basal AR total protein expression between CON and PD myoblasts (CON DM 7 ± 2 % vs. PD DM 11 ± 6 %; P = NS, Fig. 4). However, there was a significant increase in AR in testosterone treated PD myoblasts verses CON treated cells (PD T 41 ± 2 % vs. CON T 18 ± 6 %; P ≤ 0.05) after 72 h in culture. It is also worth noting this larger increase in AR with testosterone in the PD cells vs. CON cells was also shown when in the presence of IGF-IR inhibitor (PPP) (PD T + PPP 44 ± 11 % vs. CON T + PPP 13.5 ± 14.5 %; P ≤ 0.05, Fig. 4). At 7 days, testosterone also induced a larger increase in AR in PD vs. CON myoblasts (PD T 42 ± 2 % vs. CON T 15 ± 7 %; PD T + PPP 52 ± 5 % vs. CON T + PPP 12 ± 3 %; P ≤ 0.05, Fig. 4). These observations provide evidence towards a heightened response in PD myoblasts to testosterone stimulated increases in AR protein expression which may contribute to the rescuing of differentiation in fusion impaired myoblasts that display basally reduced myotube growth.

### Impact of testosterone plus AR and IGF-IR inhibitors on downstream IGF-I signaling proteins

**Fig. 5:**
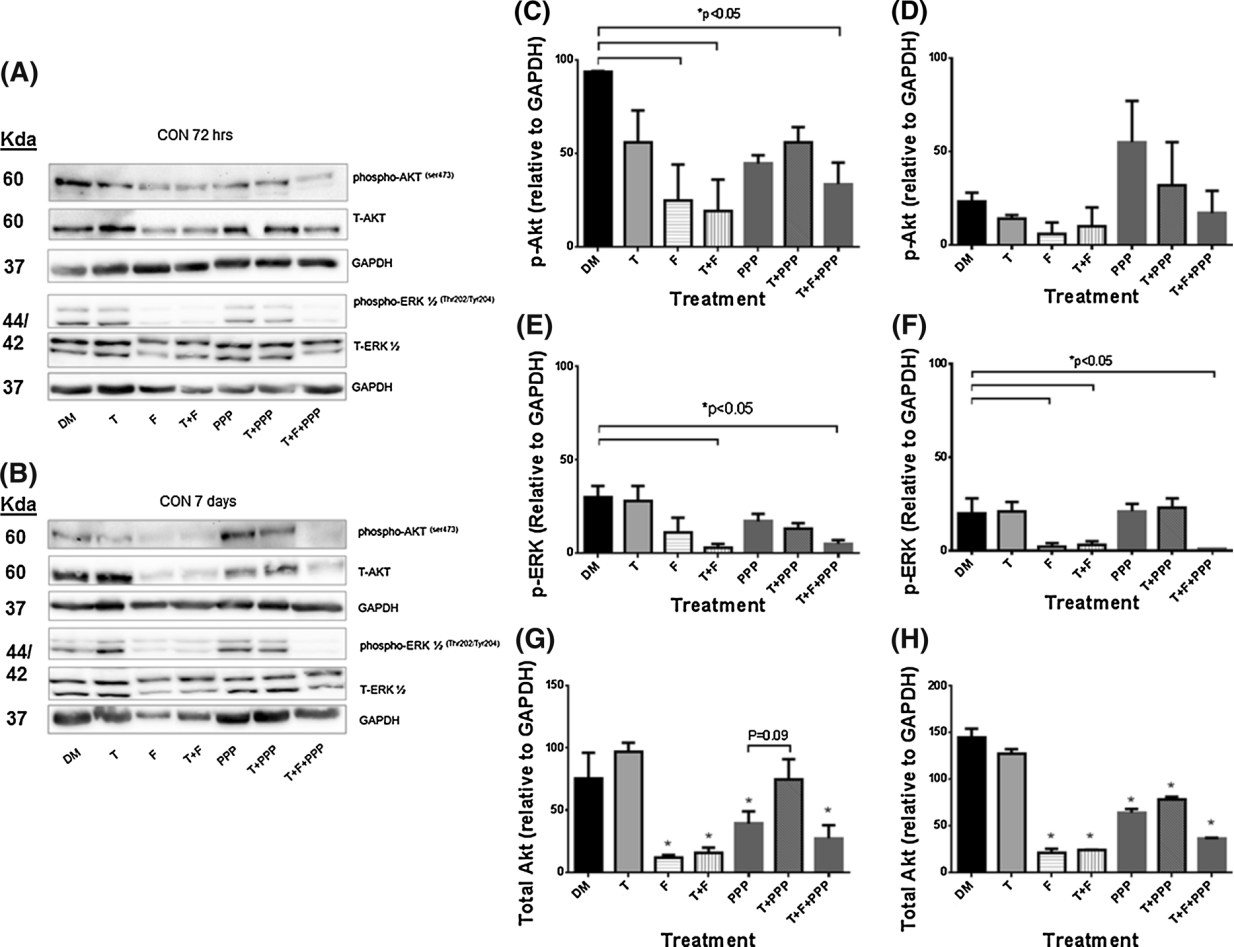
The effect of exogenous testosterone and co-incubations on total and phosphorylated Akt and ERK1/2 in CON myoblasts after 72 h (**a**, **c**, **e**, **g**) and 7 days (**b**, **d**, **f**, **h**) respectively. **a** Are representative blot images for CON cells at 72 h. **b** Are representative blot images for CON cells at 7 days. After 72 h (**c**), flutamide presence significantly reduced Akt phosphorylation compared to un-treated CON myoblast (*P < 0.05). Exogenous testosterone had no impact on alterations in Akt phosphorylation. At 7 days (**d**), there were no statistically significant alterations in phosphorylated Akt in PD myoblasts. **e** The presence of flutamide was detrimental in ERK1/2 activation (*P < 0.05). Exogenous testosterone did not significantly alter ERK1/2 activation levels. **f** Similar to 72 h, at 7 days flutamide administration continued to be detrimental for ERK1/2 phosphorylation (*P < 0.05). There were no alterations in total ERK protein at either time points. There were no differences between cell types (CON vs. PD) for any time point/condition. **g** The presence of flutamide and picropodophyllin significantly decreased total Akt protein after 72 h compared to untreated cells (*P < 0.05). The addition of testosterone with picropodophyllin lead to total Akt protein levels being similar to un-treated myoblasts and approached a significant increase compared to picropodophyllin alone (P = 0.09). **h** At 7 days, the presence of picropodophyllin and flutamide significantly reduced Akt protein levels (*P < 0.05), with testosterone having little effect in combination with the inhibitors. Values presented as Mean ± SEM

Supplementary to assessing androgen receptor protein levels with testosterone stimulus with/without AR or IGF-IR inhibitors, downstream protein activity of IGF-I signaling (Akt and ERK1/2) was investigated. Despite the IGF-IR being seemingly un-involved in the impact of testosterone on morphological measures, downstream signaling of the IGF-IR has been shown to be increased with T administration (Wu et al. 2010; Serra et al. 2011; White et al. 2012; Basualto-Alarcón et al. 2013), therefore warranted further investigation. There were no significant alterations in total ERK1/2 protein levels for either cell type or over the time course. For ERK1/2 phosphorylation, the presence of testosterone and flutamide co-incubation treatments significantly reduced levels at both 72 h (CON DM 30 ± 6 vs. T 28 ± 8; T + F 3 ± 2; T + F + PPP 5 ± 2; P ≤ 0.05, Fig. 5) and 7 days (CON DM 20 ± 8 vs. 21 ± 5; T + F 3 ± 2; T + F + PPP 1 ± 0; P ≤ 0.05, Fig. 5). In both CON and PD myoblasts, testosterone alone was ineffective in significantly increasing ERK1/2 phosphorylation at either time point. Between cell types, there were no significant differences in ERK1/2 phosphorylation basally, as reported originally in Sharples et al. (2011) or for all other treatments in the present study at either time point.

**Fig. 6:**
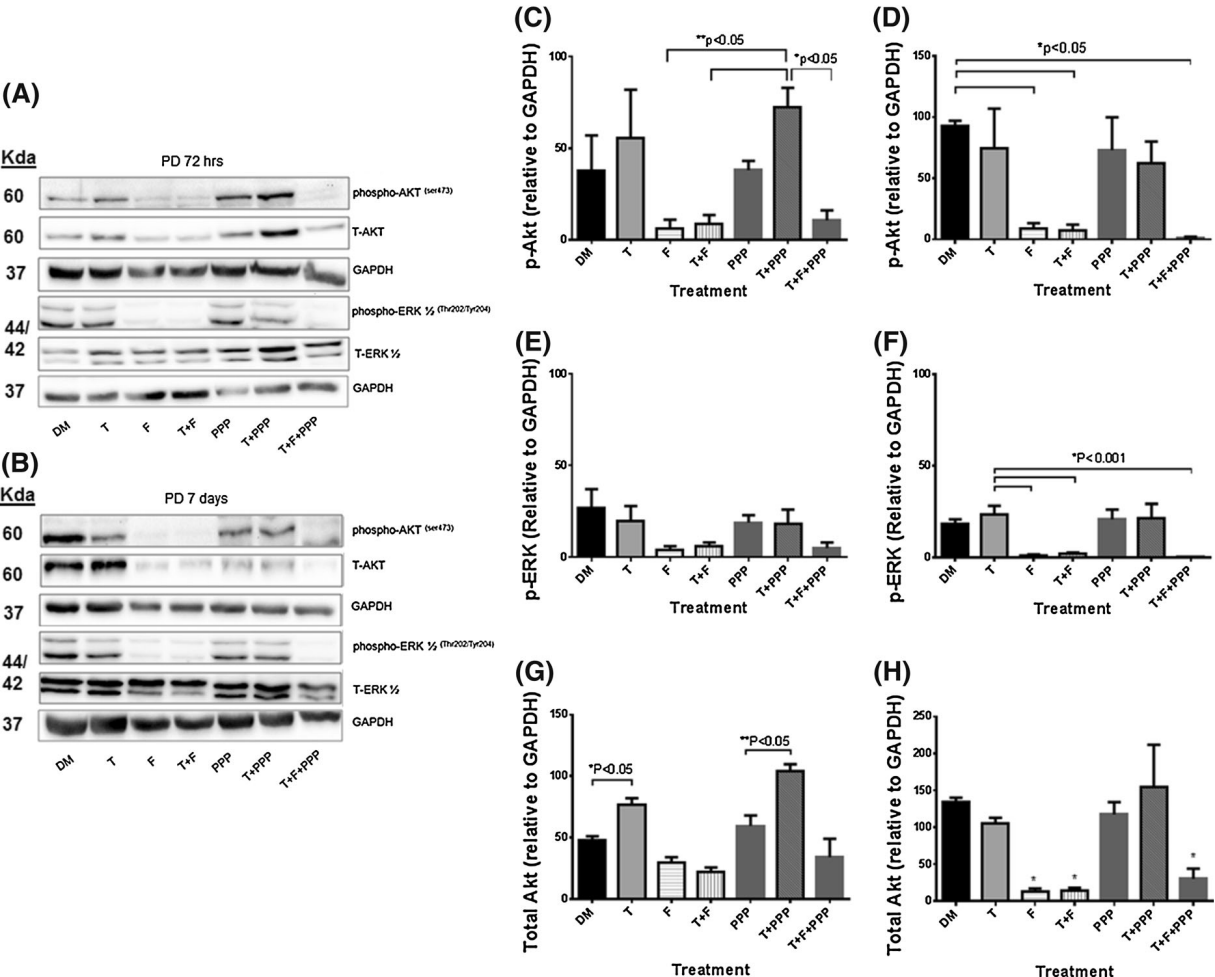
The effect of exogenous testosterone and co-incubations on total and phosphorylated Akt and ERK1/2 in PD myoblasts after 72 h (**a**, **c**, **e**, **g**) and 7 days (**b,** **d**, **f**, **h**) respectively. **a** Are representative blot images for PD cells at 72 h. **b** Are representative blot images for PD cells at 7 days. After 72 h (**c**), exogenous testosterone alone increased Akt phosphorylation (although this was not statistically significant). The co-incubation of testosterone significantly increased phosphorylated Akt levels which were reduced in the presence of flutamide (*P < 0.05). At 7 days (**d**), the presence of flutamide significantly reduced the levels of phosphorylated Akt in PD myoblasts (*P < 0.05). **e** There were no statistically significant alterations in with any treatment after 72 h, although all flutamide conditions displayed a mean trend towards reduced ERK1/2 activation. **f** At 7 days the presence of flutamide was significantly detrimental for ERK1/2 phosphorylation (*P < 0.05). **g** The addition of testosterone, even in the presence of picropodophyllin, significantly increased total Akt levels after 72 h (*P < 0.05). **h** There was no longer a significant effect of testosterone on total Akt levels after 7 days culture. The presence of flutamide significantly decreased total Akt levels compared to un-treated myoblasts (*P < 0.05). Values presented as Mean ± SEM

In CON cells, after 72 h there were significant reductions in phosporylated Akt with the presence of flutamide in all treatments (CON DM 93.5 ± 0.5 vs. F 24.5 ± 19.5; T + F 19 ± 17; T + F + PPP 33 ± 11.5; P ≤ 0.05, Fig. 5). The presence of testosterone had no effect on Akt phosphorylation at either time points in CON cells (P = N.S.). After 7 days administration, there were no statistically significant alterations with any of the treatments. In comparison, a testosterone stimulus applied to the PD myoblasts resulted in mean increases in Akt phosphorylation (although not statistically significant), after 72 h exposure. These increases were also present with the co-incubation of PPP, yet the presence of flutamide significantly reduced Akt phosphorylation (PD T + PPP 72.5 ± 10; F 6 ± 5; T + F 8.5 ± 5; T + F + PPP 10.5 ± 5.5; P ≤ 0.05, Fig. 6). At 7 days, the average (but not significant) increases in Akt phosphorylation with a testosterone stimulus were no longer present in PD myoblasts. However, the presence of flutamide was still detrimental to Akt phosphorylation in PD cells (PD DM 93 ± 4 vs. F 9 ± 4; T + F 7 ± 5; T + F + PPP 1 ± 1; P ≤ 0.05, Fig. 6).

Total Akt protein significantly decreased in the presence of flutamide and picropodophyllin after 72 h in the CON cells. The co-incubation of testosterone and PPP lead to total Akt levels increasing compared to PPP alone (CON T + PPP 74.5 ± 16.5 vs. PPP 39 ± 10; P = 0.09, Fig. 5), back to levels observed in un-treated CON myoblasts at 72 h (CON DM 75 ± 21). After 7 days culture, statistically significant reductions remained for total Akt levels in all treatments with PPP and flutamide (CON DM 144 ± 10 % vs. F 20.5 ± 5; PPP 64 ± 4; T + F + PPP 36 ± 1; P ≤ 0.05, Fig. 5). In comparison, the presence of testosterone significantly increased total Akt protein after 72 h culture in PD myoblasts, with this observation being abrogated in the presence of flutamide (PD DM 47.5 ± 3.5 vs. T 76.5 ± 5.5; T + F 22 ± 4; P ≤ 0.05, Fig. 6). It therefore appears that testosterone administration increases abundance of Akt protein levels in both cell types, which may subsequently influence further downstream signaling involved in the restored myotube morphology observed in PD cells, albeit independently of the IGF-I receptor.

Additionally, between cell types, there were significant differences in Akt phosphorylation for basal treatments in CON and PD myoblasts, where there was a significant reduction after 72 h in PD myoblasts (CON DM 93.5 ± 0.5 vs PD DM 37.5 ± 19.5; P ≤ 0.01). However at 7 days, Akt phosphorylation was significantly increased in PD myoblasts compared to basal CON cells highlighting a delayed response and may explain the delay in fusion within this cell type (PD DM 93 ± 5 vs. CON DM 23 ± 5; P ≤ 0.001). For all other treatments between the cell types, no significant differences were observed at any time point.

Overall, the alterations in cellular signaling provide evidence towards a functional AR receptor being required for normal myotube formation and hypertrophy in control and PD myoblasts, as flutamide reduces these parameters with associated reductions in phosphorylation of ERK and Akt. Importantly, testosterone alone appeared to have a significantly larger impact on Akt abundance in the PD myoblasts vs. CON cells with little effect being observed in ERK1/2 activity and abundance following  testosterone administration in either cell type.

### Effects of testosterone administration on gene transcripts in control and PD myoblasts

**Fig. 7:**
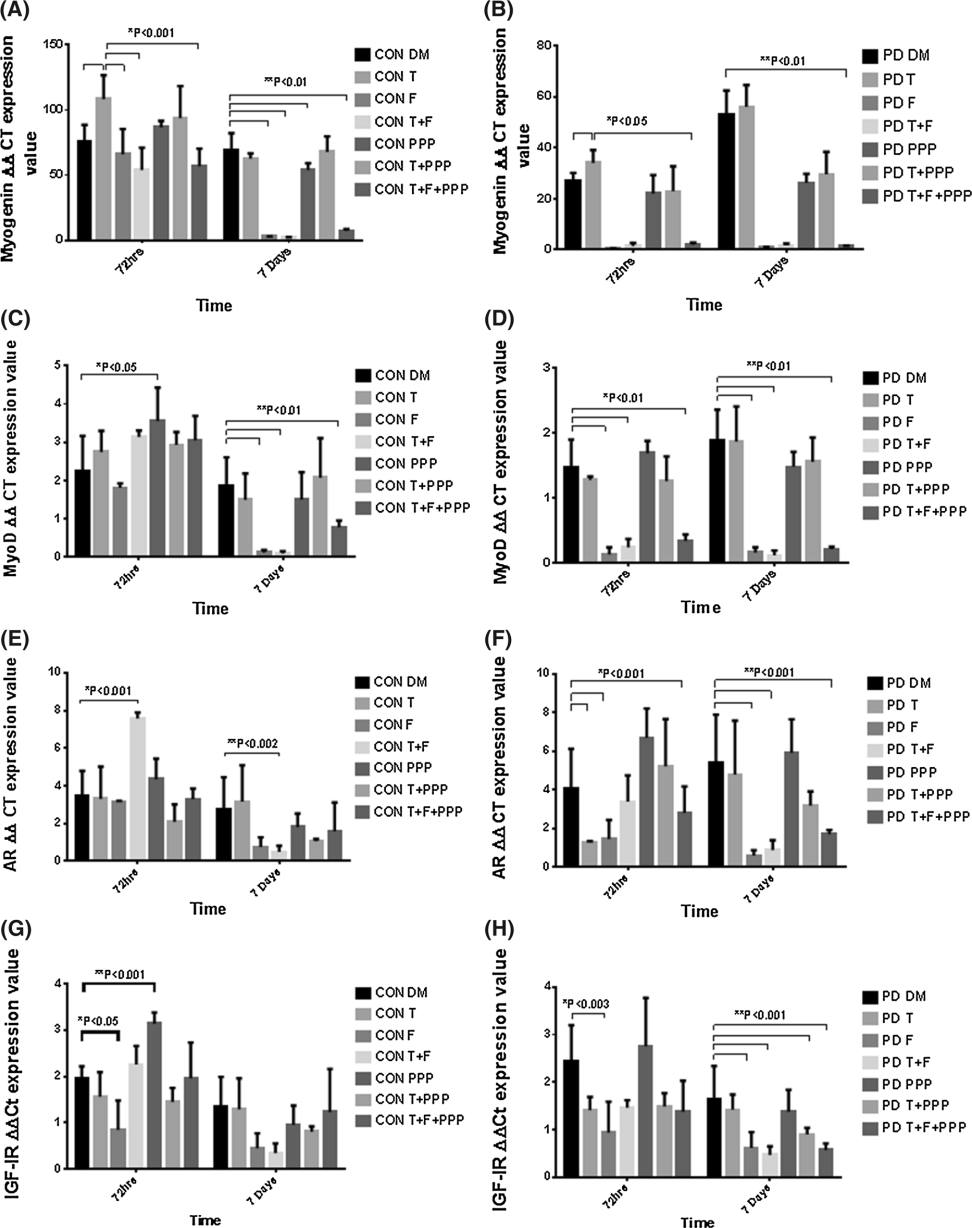
Impact of exogenous testosterone and AR (Flutamide/F)/IGF-IR (picropodophyllin/PPP) inhibitor co-incubations on MyoD, myogenin, AR and IGF-IR mRNA in CON and PD myoblasts after 72 h and 7 days. **a** Picropodophyllin alone significantly increased MyoD levels after 72 h (*P < 0.05). At 7 days, the presence of flutamide reduced MyoD expression levels in CON myoblasts (**P < 0.01). **b** In PD myoblasts, testosterone had no impact on MyoD levels at either time point. The presence of flutamide significantly reduced expression levels after 72 h (*P < 0.01) and 7 days (**P < 0.01). **c** Testosterone treatment significantly increased myogenin expression levels in CON myoblasts after 72 h (*P < 0.001). The presence of flutamide significantly reduced testosterone induced increases in myogenin (*P < 0.001). The presence of PPP did not significantly reduce increases in myogenin under a testosterone stimulus after 72 h culture. **d** In PD myoblasts, testosterone administration increased myogenin levels (P < 0.05) compared to un-treated cells. This observation was abrogated by the presence of flutamide and Picropodophyllin (*P < 0.05). At 7 days, there were no increases in myogenin expression with testosterone, however the presence of flutamide resulted in reduced levels (**P < 0.01). There was a significant difference between myogenin mRNA expression after 72 h and 7 days in untreated PD cells (***P < 0.01). **e** Testosterone co-incubated with flutamide significantly increased AR mRNA expression levels after 72 h in CON myoblasts (*P < 0.001). This effect was significantly reduced after 7 days (**P < 0.002). **f** In PD myoblasts, there significant reductions in AR expression with testosterone alone and in the presence of flutamide after 72 h (*P < 0.001). At 7 days, the addition of flutamide alone resulted in AR expression remaining decreased (**P < 0.001). **g** In CON myoblasts, flutamide significantly reduced IGF-IR expression (*P < 0.05) and Picropodophyllin significantly increased IGF-IR at the same time point (**P < 0.001). There were no observed alterations at 7 days exposure. **h** Flutamide significantly reduced IGF-IR expression after 72 h in PD myoblasts compared to un-treated cells (*P < 0.003). The presence of flutamide, after 7 days treatment, resulted in significant reductions in IGF-IR expression compared to basal conditions (**P < 0.01). Values presented as Mean ± SD

Gene transcripts were analyzed to determine the impact of treatments on selected genes involved in muscle differentiation and myotube hypertrophy in both CON and PD myoblasts. Testosterone significantly increased myogenin mRNA expression in both CON (DM 75.86 ± 12.67 vs. T 108.36 ± 18.34; P ≤ 0.001, Fig. 7a) and PD (DM 27.03 ± 3.02 vs. T 34.05 ± 5.03; P ≤ 0.05, Fig. 7b) myoblasts after 72 h exposure. After 7 days exposure, the observed increases in myogenin with testosterone treatment were no longer present in either cell type (P = NS). In basal conditions, CON cells had significantly higher expression of myogenin after 72 h compared to PD myoblasts (CON DM 75.86 ± 12.67 vs. PD DM 27.03 ± 3.02; P ≤ 0.01, Fig. 7b), with this effect not observed at 7 days (P = NS, Fig. 7b). The administration of testosterone had no impact in either cell type on expression of MyoD, IGF-I receptor and myostatin (P = NS). In PD myoblasts, testosterone treatment significantly reduced AR mRNA levels after 72 h exposure (DM 4.07 ± 2.04 vs. T 1.28 ± 0.05; P ≤ 0.001, Fig. 7f), which is an opposite observation to changes in AR at the protein level described above.

### Flutamide administration significantly reduced gene transcription levels

Flutamide was inhibitory, particularly for transcripts involved in muscle differentiation (MyoD and Myogenin). After 7 days exposure, myogenin expression was significantly decreased in CON cells with the presence of flutamide in all treatments compared to basal conditions (CON DM 69.07 ± 13.15 vs. F 2.84 ± 0.11; T + F 2.06 ± 0.33; T + F + PPP 6.99 ± 1.54; P ≤ 0.01, Fig. 7a). The detrimental effect with flutamide administration in all treatments on myogenin expression was observed in PD myoblasts after 72 h exposure (PD DM 34.05 ± 5.03 vs. F 0.30 ± 0.11; T + F 1.44 ± 1.14; T + F + PPP 1.83 ± 0.91; P ≤ 0.01, Fig. 7b) and 7 days (PD DM 56.04 ± 8.50 vs. F 1.02 ± 0.08; T + F 1.60 ± 0.73; T + F + PPP 1.39 ± 0.10; P ≤ 0.01, Fig. 7b).

Between cell types, PD myoblasts appeared to be more susceptible to flutamide treatment than CON cells. Although there was no impact of flutamide at 72 h, significant reductions in myoD expression were observed in CON cells at 7 days with the presence of flutamide in all treatments (CON DM 1.88 ± 0.73 vs. F 0.12 ± 0.07; T + F 0.10 ± 0.06; T + F + PPP 0.78 ± 0.18; P ≤ 0.01, Fig. 7c). A similar pattern occurred for myoD mRNA in the PD myoblasts after 72 h (PD DM 1.48 ± 0.42 vs. F 0.13 ± 0.11; T + F 0.24 ± 0.13; T + F + PPP 0.34 ± 0.10; P ≤ 0.01, Fig. 7d) and 7 days (PD DM 1.88 ± 0.48 vs. F 0.16 ± 0.08; T + F 0.12 ± 0.07; T + F + PPP 0.21 ± 0.04; P ≤ 0.01, Fig. 7d) following exposure to flutamide. MyoD expression was significantly reduced in PD myoblasts administered with flutamide compared to the same condition in CON myoblasts (PD + F 0.13 ± 0.11 vs. CON + F 1.81 ± 0.12; P ≤ 0.05) after 72 h culture. A similar trend for MyoD expression was observed in both testosterone plus flutamide (PD T + F 0.24 ± 0.13 vs. CON T + F 3.14 ± 0.17; P ≤ 0.05) and PPP (PD T + F + PPP 0.34 ± 0.10 vs. CON T + F + PPP 3.05 ± 0.64; P ≤ 0.05) co-incubations after 72 h culture. Furthermore, the alterations in myogenin expression, supports the increased susceptibility of PD myoblasts to flutamide administration. Myogenin expression was significantly decreased after 72 h in PD myoblasts with flutamide compared to CON treated myoblasts (PD + F 0.30 ± 0.11 vs. CON + F 65.95 ± 19.37; P ≤ 0.05). This pattern was further observed at 72 h in treatments where flutamide was present (PD T + F 1.44 ± 1.14 vs. CON T + F 53.98 ± 16.97; PD T + F + PPP 1.83 ± 0.91 vs. CON T + F + PPP 56.68 ± 13.65; P ≤ 0.05). Overall, the presence of flutamide completely abolishes MyoD and myogenin expression after 72 h and 7 days in PD myoblasts, regulatory factors which are fundamental in the fusion of myoblasts.

In CON cells, AR expression was significantly increased when co-incubated with testosterone after 7 days exposure (CON DM 2.76 ± 1.69 vs. T 3.16 ± 1.93; T + F 0.49 ± 0.32; P ≤ 0.002, Fig. 7e). To note, the administration of PPP significantly increased AR mRNA expression after 72 h exposure (CON DM 2.76 ± 1.69 vs. PPP 4.35 ± 1.09; P ≤ 0.001, Fig. 7e). In the PD myoblasts, both the presence of testosterone and flutamide significantly reduced AR mRNA after 72 h (PD DM 4.07 ± 2.04 vs. T 1.28 ± 0.05; F 1.44 ± 0.99; T + F + PPP 2.79 ± 1.37; P ≤ 0.001, Fig. 7f). For 7 days, the presence of flutamide in treatments significantly reduced AR expression (PD DM 5.42 ± 2.46 vs. F 0.59 ± 0.27; T + F 0.87 ± 0.51; T + F + PPP 1.70 ± 0.21; P ≤ 0.001, Fig. 7f).

IGF-I receptor mRNA was significantly reduced with flutamide administration after 72 h exposure in CON cells (CON DM 1.96 ± 0.26 vs. F 0.85 ± 0.63; P ≤ 0.05, Fig. 7g). The addition of PPP significantly increased IGF-I receptor expression in CON cells at 72 h (CON DM 1.96 ± 0.26 vs. PPP 3.14 ± 0.24; P ≤ 0.001, Fig. 7g). The presence of flutamide significantly reduced IGF-I receptor expression in PD myoblasts after 7 days exposure (PD DM 1.65 ± 0.69; F 0.62 ± 0.33; T + F 0.47 ± 0.18; T + F + PPP 0.57 ± 0.14; P ≤ 0.001, Fig. 7h). Furthermore, testosterone co-incubated with PPP decreased IGF-I receptor mRNA expression after 7 days (PD DM 1.65 ± 0.69 vs. T + PPP 0.90 ± 0.14; P ≤ 0.001, Fig. 7h).

**Fig. 8:**
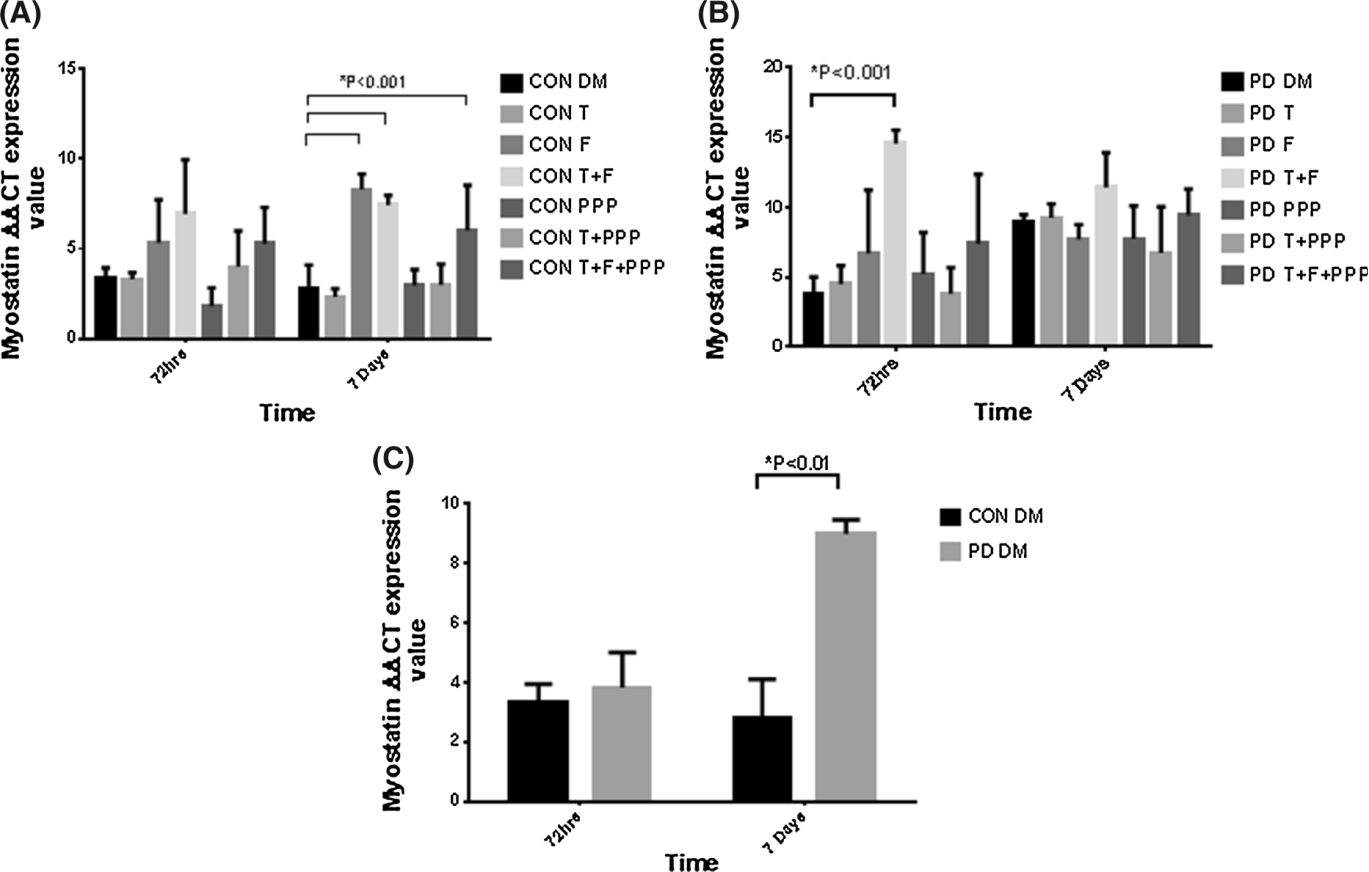
The effect of testosterone administration and AR/IGF-I inhibitor co-incubations on myostatin mRNA levels in CON and PD myoblasts after 72 h and 7 days. **a** There were no statistically significant differences in myostatin levels between treatments in CON myoblasts after 72 h. At 7 days, there were sizeable increases in myostatin levels with flutamide present compared to basal conditions (*P < 0.001). **b** After 72 h, the co-incubation of testosterone and flutamide increased myostatin significantly compared to un-treated PD cells (P < 0.001). At 7 days, there were no statistically different changes between treatments in PD myoblasts. **c** There was a significant increase in myostatin mRNA levels after 7 days culture between basal CON and PD myoblasts (*P ≤ 0.01). Values presented as Mean ± SD

Finally, as myostatin has recently been shown to be responsive to androgens (Dubois et al. 2014), the reported observations in myotube morphology with flutamide administration may be underpinned by the alterations in myostatin mRNA levels. The presence of flutamide significantly increased myostatin expression after 7 days exposure in CON myoblasts (CON DM 2.78 ± 1.32 vs. F 8.30 ± 0.85; P ≤ 0.001, Fig. 8a). In PD myoblasts, treatments containing flutamide increased myostatin expression at both time points, although this was not statistically significant. After 72 h, flutamide co-incubated with testosterone significantly increased myostatin expression in PD myoblasts (PD DM 3.83 ± 1.17 vs. F 6.70 ± 4.51; T + F 11.39 ± 2.49; P ≤ 0.001, Fig. 8b). In this experiment, testosterone and PPP had no impact on myostatin expression in either cell type. There were significant differences in myostatin expression between basal CON and PD myoblasts after 7 days culture (CON DM 2.78 ± 1.32 vs. PD DM 8.99 ± 0.47; P ≤ 0.01, Fig. 8c). Myostatin has been observed to be detrimental in myotube size and inhibiting muscle differentiation, thus potentially explaining the lack of myotube formation in the current study, with AR inhibition (Trendelenburg et al. 2009; Dubois et al. 2014).

## Discussion

### Exogenous testosterone administration restores an ageing phenotype via the androgen receptor and Akt

The current studies aim (1) sought to investigate the predominate pathway for mediating testosterone induced hypertrophy as commonly the AR and IGF-I pathway have been investigated independently (Wu et al. 2010; Serra et al. 2011; White et al. 2012). The alterations in myotube morphology and protein signaling (discussed below) provide evidence towards a pivotal role for a functional androgen receptor being required in myotube formation and hypertrophy in control (CON) and fusion impaired myoblasts that display myotube atrophy (PD) and thus the original hypothesis for both AR and IGF-IR being pivotal in testosterone induced hypertrophy was rejected. As we have previously published the ability of testosterone to improve impaired fusion and myotube atrophy in the PD myoblasts via increases myotube diameter (Deane et al. 2013), we sought to further elucidate the cellular and molecular mechanisms for the positive alterations observed in the PD myoblast phenotype. The positive effect of a testosterone stimulus on myotube morphology in CON and PD myoblasts was accompanied by greater myogenin expression, similar to previous literature (Lee 2002; Wannenes et al. 2008). At the transcriptional level, exogenous testosterone had little impact on other genes investigated, although it resulted in AR mRNA being significantly down-regulated in the PD myoblasts after 72 h culture, in contrast to the concomitant increases observed in total AR protein. This observation may be hypothesized to be due to a potential increase in T-AR interactions at the protein level, leading to reduced AR transcriptional activity, however this requires further investigation. Also, the ability of testosterone to significantly increase androgen receptor levels in PD myoblasts persisted even in the presence of the IGF-I inhibitor (picropodophyllin). The AR increases in PD myoblasts were significantly greater compared to testosterone stimulated CON myoblasts and provide evidence for the involvement of T in rescuing the impaired differentiation and myotube atrophy via increases in the abundance of the androgen receptor. This was accompanied via increases in myogenin gene expression, furthermore when the AR was inhibited, reductions in MyoD occurred, suggesting that androgen receptor was fundamental in both the reductions in differentiation and hypertrophy evident when incubated in the presence of flutamide alone, and in testosterone induced hypertrophy. Recently, Serra et al. (2011) highlighted the role of IGF-I signaling in mediating the effects of testosterone on skeletal muscle mass. The authors noted that a testosterone stimulus was mediated via IGF-I/IGF-IR and downstream signaling. The inhibition of IGF-IR was achieved through small interference RNA compared to the current study which utilized a substrate competitor inhibitor (Picropodophyllin). Contrary to the findings of Serra et al. (2011) we observed testosterone-induced hypertrophy in both cell types even in the presence of the IGF-I receptor inhibitor, supported by the findings of Wu et al. (2010). However, it is worth noting that reductions in Akt and ERK activity were only approximately 40 % in the present studies with PPP. The most recent evidence has alluded to the potential role of Akt/mTOR signaling in testosterone induce hypertrophy (White et al. 2012; Basualto-Alarcón et al. 2013). Within the current study, increases in downstream IGF-IR signaling (total Akt) were apparent in the PD myoblasts only with testosterone administration after 72 h culture, an observation that continued even in the presence of the IGF-IR inhibitor (PPP). PD myoblasts also had basally reduced phosphorylated Akt at key time points in differentiation (72 h) versus control myoblasts and recently we demonstrated Akt inhibition (using LY294002) has been observed to abrogate testosterone-induced myotube hypertrophy (Deane et al. 2013). It is important to note that acute increases in Akt phosphorylation may have occurred earlier under a testosterone stimulus, as previously reported (Basualto-Alarcón et al. 2013). However, the increase in Akt abundance in both cells types and increased abundance in PD versus CON cells, allied to its sustained elevation despite IGF-IR inhibition provides supportive evidence for the interaction between testosterone and Akt perhaps somewhat independently of the IGF-I receptor. Having said this, it is important to note that in AR inhibitor alone conditions, flutamide reduced IGF-IR mRNA in both cell types across time points, with an observed reduction in activity of ERK and Akt, suggesting that IGF-IR is transcriptionally regulated by AR and therefore its downstream effector pathway. However, where testosterone increased AR protein content there was no increases observed in IGF-IR gene expression. This suggested that sufficient AR was important to enable normal IGF-IR gene expression and downstream signaling, yet elevated levels of AR due to testosterone had no further effect on IGF-IR, despite testosterone increasing Akt abundance in the presence of IGF-IR inhibitor (discussed above).

### The critical role of the androgen receptor in skeletal myotube hypertrophy

The androgen receptor has been observed to be important in skeletal muscle development and muscle differentiation (Wannenes et al. 2008), as the knockout of muscle-specific AR results in reduced muscle mass and strength (MacLean et al. 2008). In the present study, flutamide (AR antagonist) administration alone completely blocked myotube formation in both CON and PD myoblasts. As aluded to above the presence of flutamide significantly reduced IGF-IR mRNA and Akt and ERK1/2 activation in both cell types which provides evidence that IGF-IR is transcriptionally regulated by AR and that basally (without testosterone) AR is important in normal Akt and ERK activity important in cellular differentiation and myotube hypertrophy (Baar and Esser 1999; Jones et al. 2001; Rommel et al. 2001; Adi et al. 2002). Importantly, the presence of flutamide abrogated testosterone induced hypertrophy in both cell types, suggesting AR being a critical pathway for the improvements in myotube hypertrophy under a testosterone stimulus. Furthermore, in both CON and PD myoblasts with the presence of flutamide, there was a significant increase in myostatin mRNA expression. Myostatin (GDF-8; TGF-β family member) has been observed to be a negative regulator of muscle mass and more specifically inhibit muscle differentiation and myotube size (McCroskery et al. 2003; Amthor et al. 2004; Trendelenburg et al. 2009). Recently Braga et al. (2012), reported the effects of testosterone on TGF-β signaling where testosterone stimulated muscle cell proliferation and differentiation due to inhibition of this pathway and via follistatin up-regulation. Furthermore, Dubois et al. (2014) have highlighted promotion of myostatin gene expression as a direct target for androgen receptor mediated transcription through studies investigating satellite-cell specific AR knockout mice (satARKO). The authors found that satARKO mice had reduced muscle strength, yet because of AR’s transcription factor role in myostatin expression, the authors  actually saw a sixfold reductions in myostatin. This perhaps shows that AR maybe even be more powerful in controlling muscle mass than myostatin because there was still a drop in strength in satARKO mice but the corresponding drop in myostatin could not  compensate in order to increase strength. Indeed, this was confirmed where muscle hypertrophy in response to androgens was enhanced in myostatin knockout mice. This conflicts with the findings of the current study, where the inhibition of the AR lead to increased myostatin levels, leading to reduced myotube differentiation, however this phenotype has been previously observed in human muscle cells with the addition of myostatin (Trendelenburg et al. 2009). In the current study, exogenous testosterone had no positive effect on myostatin expression in either cell type, although previous animal and in vitro studies have observed testosterone-induced reductions in myostatin levels (Kawada et al. 2006; Mendler et al. 2007; Kovacheva et al. 2010; Deane et al. 2013), findings that warrant future investigation in this model. There is also evidence that myostatin inhibits satellite cell activation, proliferation, and differentiation (Wagner et al. 2005; Kawada et al. 2006; Sinha-Hikim et al. 2006), through perturbation of Akt and mTOR signalling (Trendelenburg et al. 2009). In the current study, Akt abundance was increased with a testosterone stimulus in PD cells and reduced in the presence of flutamide. Therefore, the reduced Akt in the flutamide conditions may have been as a consequence of the increased myostatin levels observed. Thus administration of testosterone shows strong potential to enhance hypertrophy in fusion impaired myoblasts that display myotube atrophy directly via the AR, however also highlights the importance of total Akt abundance. Indeed, previous literature has implicated testosterone enhancing Akt/mTOR pathway signalling (Wu et al. 2010; White et al. 2012; Basualto-Alarcón et al. 2013) and previously we have provided evidence towards its role in aged myoblasts (Deane et al. 2013). Therefore the regulation of muscle hypertrophy under a testosterone stimulus appears to be controlled by AR, IGF-IR, Akt and myostatin interactions. Future work needs to address the mechanisms of increased alterations in Akt abundance following testosterone administration despite blocking upstream IGF-IR.

## Conclusion

In conclusion, testosterones ability to improve differentiation and myotube hypertrophy occurred predominately via increases in AR and Akt abundance in both CON and PD cells, with fusion impaired cells (PD) showing an increased responsiveness to T induced AR levels. Finally, T induced increases in myotube hypertrophy (but not early differentiation) occurred independently of upstream IGF-IR input, however it was apparent that normal AR function in basal conditions (without testosterone) was required for adequate IGF-IR gene expression and downstream ERK/Akt activity.
